# Prolonged ventricular repolarization associated with mild cognitive impairment and white matter hyperintensities: a cross-sectional study

**DOI:** 10.1038/s41598-024-65364-y

**Published:** 2024-07-02

**Authors:** Ming Mao, Yiran Wei, Chaoqun Wang, Xiaolei Han, Rui Liu, Yi Dong, Lin Song, Lin Cong, Yongxiang Wang, Yifeng Du, Chengxuan Qiu

**Affiliations:** 1 Department of Neurology, Shandong Provincial Hospital affiliated to Shandong First Medical University, No. 324 Jingwuweiqi Road, Jinan, 250021 Shandong People’s Republic of China; 2 Key Laboratory of Endocrine Glucose and Lipids Metabolism and Brain Aging in Shandong First Medical University, Ministry of Education of the People’s Republic of China, Jinan, 250021 Shandong People’s Republic of China; 3 Department of Neurology, Shandong Provincial Hospital, Shandong University, Jinan, 250021 Shandong People’s Republic of China; 4 Institute of Brain Science and Brain-Inspired Research, Shandong First Medical University and Shandong Academy of Medical Sciences, Jinan, 250021 Shandong People’s Republic of China; 5 Department of Neurobiology, Care Sciences and Society, Aging Research Center and Center for Alzheimer Research, Karolinska Institutet-Stockholm University, 17177 Stockholm, Sweden

**Keywords:** Prolonged ventricular repolarization, Mild cognitive impairment, Electrocardiogram, White matter hyperintensities, Cross-sectional study, Cognitive ageing, Cardiovascular diseases, Epidemiology, Alzheimer's disease, Psychology

## Abstract

Prolonged ventricular repolarization has been associated with cardiovascular disease. We sought to investigate the association of prolonged ventricular repolarization with mild cognitive impairment (MCI) and the potential underlying neuropathological mechanisms in older adults. This cross-sectional study included 4328 dementia-free participants (age ≥ 65 years; 56.8% female) in the baseline examination of the Multidomain INterventions to delay dementia and Disability in rural China; of these, 989 undertook structural brain magnetic resonance imaging (MRI) scans. QT, QTc, JT, JTc, and QRS intervals were derived from 12-lead electrocardiograph. MCI, amnestic MCI (aMCI), and non-amnestic MCI (naMCI) were defined following the Petersen’s criteria. Volumes of gray matter (GM), white matter, cerebrospinal fluid, total white matter hyperintensities (WMH), periventricular WMH (PWMH), and deep WMH (DWMH) were automatically estimated. Data were analyzed using logistic and general linear regression models. Prolonged QT, QTc, JT, and JTc intervals were significantly associated with an increased likelihood of MCI and aMCI, but not naMCI (*p* < 0.05). In the MRI subsample, QT, QTc, JT, and JTc intervals were significantly associated with larger total WMH and PWMH volumes (*p* < 0.05), but not with DWMH volume. Statistical interactions were detected, such that prolonged QT and JT intervals were significantly associated with reduced GM volume only among participants with coronary heart disease or without *APOE* ε4 allele (*p* < 0.05). Prolonged ventricular repolarization is associated with MCI and cerebral microvascular lesions in a general population of older adults. This underlies the importance of cognitive assessments and brain MRI examination among older adults with prolonged QT interval.

## Introduction

Mild cognitive impairment (MCI) is considered a transitional stage between normal cognitive aging and dementia, and people with MCI show an increased risk of progression to dementia^1^. MCI can be subdivided into amnestic MCI (aMCI) and non-amnestic MCI (naMCI), based on the presence of memory impairment, where aMCI is more commonly associated with progression to Alzheimer's dementia (AD)^2^. Accumulated evidence has shown that cardiometabolic risk factors and related cardiovascular disease (CVD) are tightly associated with cognitive impairment^3–5^, partially due to common pathophysiological mechanisms (e.g., arterial stiffness, thrombo-embolism, and cerebral hypoperfusion)^6^. The ventricular electrocardiogram (ECG) profiles are the early markers of cardiovascular disorders. For example, QT interval and JT interval (QT-QRS) reflect ventricular repolarization, while QRS interval indicates ventricular depolarization. Besides, a prolonged QT interval was associated with coronary heart disease (CHD), impaired right ventricular function, and clinical stroke^7–9^, suggesting that altered ventricular repolarization may have an impact on the brain, and thus, cognitive function. Previous studies have linked prolonged QT interval with poor cognitive function^10,11^, but the community-based studies that explore the relationships of prolonged ventricular repolarization with MCI and its subtypes in older adults are currently lacking.

The underlying neuropathological pathways linking ECG markers to cognitive phenotype are poorly characterized. Data from the Prospective Study of Pravastatin in the Elderly at Risk (PROSPER) showed no associations of prolonged ventricular repolarization with any of the examined brain MRI metrics (e.g., grey matter volume, number of microbleeds, and white matter hyperintensities [WMH]) in older individuals with high cardiovascular risk^12^. However, whether prolonged QT and JT intervals are associated with structural brain lesions in a general population of older adults is unknown.

**Figure 1:**
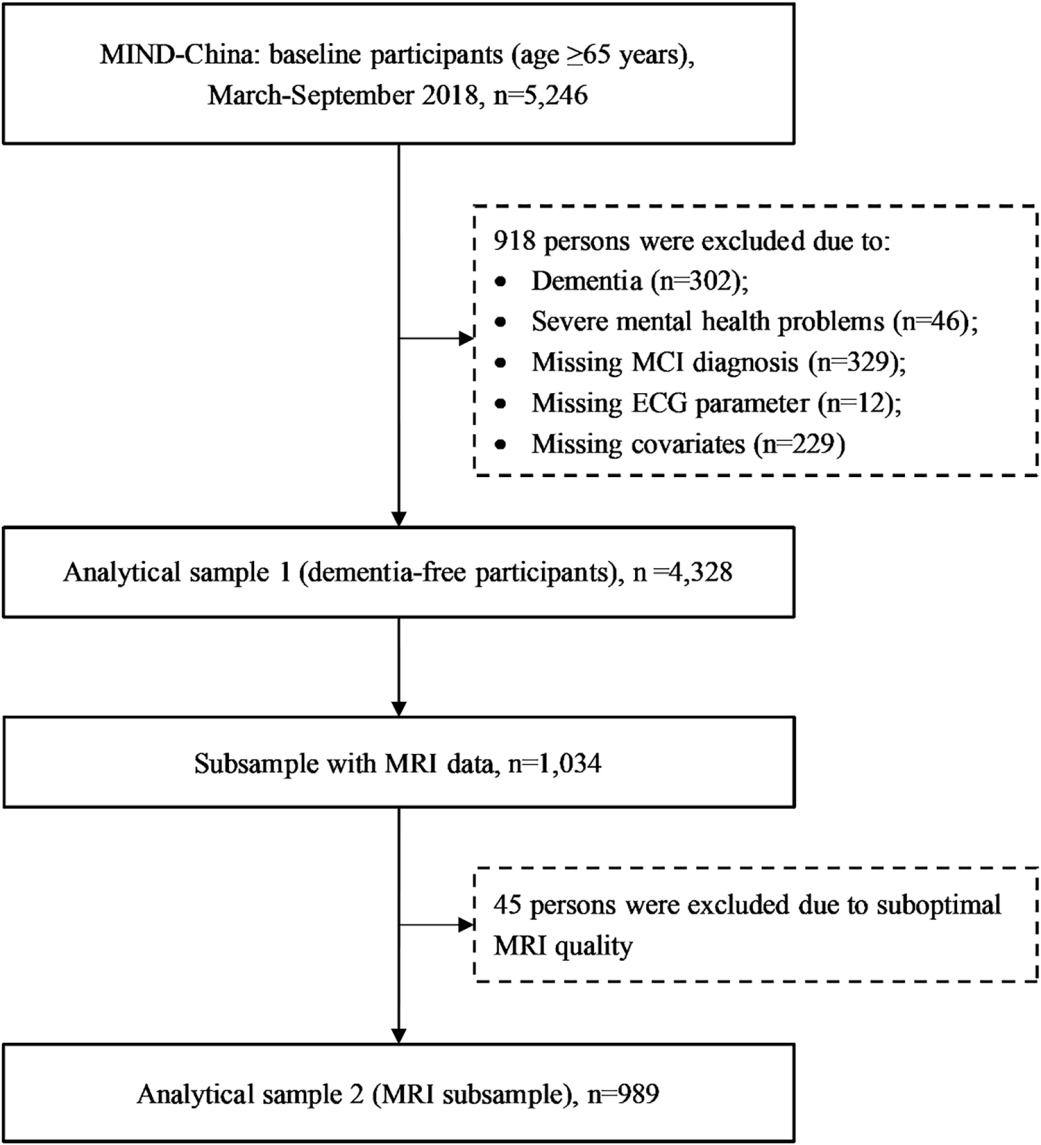
Flowchart of the study participants. MIND-China, multimodal interventions to delay dementia and disability in rural China; MCI, Mild cognitive impairment; ECG Electrocardiogram; MRI, Magnetic resonance imaging.

In this cross-sectional study, we sought to examine the association of QT, QTc, JT, JTc, and QRS intervals with MCI, subtypes of MCI, and brain MRI metrics in rural older adults in China. Our hypothesis was that prolonged ventricular repolarization was associated with structural brain lesions, and thus, with MCI, in old age (Fig. 1).

## Results

### *Characteristics of study participants (n* = *4328)*

The mean age of the 4,328 participants was 71.02 years (age range, 65–91 years; standard deviation [SD], 4.7 years), 56.8% were female, and 37.4% had no formal education. Out of these, 1172 (27.1%) were diagnosed with MCI, including 991 with aMCI and 181 with naMCI. Compared to cognitively normal participants (n = 3156), those with MCI were older; more likely to be female; less educated; had a higher systolic blood pressure and resting heart rate, lower eGFR and BMI, longer QTc, JT, and JTc intervals, shorter QRS interval; and had a higher prevalence of stroke (*p* < 0.05, Table 1).

**Table 1:** Characteristics of study participants by mild cognitive impairment.

Characteristics	Total sample	Mild cognitive impairment		
	(n = 4328)	No (n = 3156)	Yes (n = 1172)	*P-*value
Age, years	71.02 (4.7)	70.74 (4.6)	71.79 (5.1)	< 0.001
Female sex	2460 (56.8)	1679 (53.2)	781 (66.6)	< 0.001
Education, n (%)				< 0.001
No formal education	1618 (37.4)	1048 (33.2)	570 (48.6)	
Primary school	1978 (45.7)	1458 (46.2)	520 (44.4)	
Middle school or above	732 (16.9)	650 (20.6)	82 (7.0)	
Smoking status, n (%)				< 0.001
Never	2764 (63.9)	1920 (60.8)	844 (72.0)	
Former	656 (15.2)	516 (16.3)	140 (11.9)	
Current	908 (21.0)	720 (22.8)	188 (16.0)	
Alcohol intake, n (%)				< 0.001
Never	2642 (61.0)	1808 (57.3)	834 (71.2)	
Former	409 (9.5)	298 (9.4)	111 (9.5)	
Current	1277 (29.5)	1050 (33.3)	227 (19.4)	
SBP, mmHg *	143.88 (21.4)	143.33 (20.9)	145.36 (22.6)	0.01
DBP, mmHg *	85.08 (10.9)	85.11 (10.9)	85.00 (10.9)	0.61
TC, mmol/l	4.99 (1.0)	4.99 (1.0)	5.01 (1.0)	0.52
eGFR, (ml/min/1.73m^2^)	78.86 (12.9)	79.28 (12.5)	77.72 (13.7)	0.006
BMI, kg/m^2^ a	24.92 (3.8)	25.00 (3.7)	24.70 (3.9)	0.020
Diabetes mellitus, n (%)	603 (13.9)	440 (13.9)	163 (13.9)	> 0.99
Dyslipidemia, n (%)	1005 (23.2)	725 (23.0)	280 (23.9)	0.55
Hypertension, n (%)	2901 (67.0)	2099 (66.5)	802 (68.4)	0.247
Coronary heart disease, n (%)	931 (21.5)	673 (21.3)	258 (22.0)	0.65
Heart failure, n (%)	130 (3.0)	96 (3.0)	34 (2.9)	0.89
Arrhythmia, n (%)	604 (14.0)	454 (14.4)	150 (12.8)	0.20
Stroke, n (%)	647 (14.9)	431 (13.7)	216 (18.4)	< 0.001
TIAs, n (%)	50 (1.2)	36 (1.1)	14 (1.2)	> 0.99
Anti-thrombotic agents, n (%)	281 (6.5)	215 (6.8)	66 (5.6)	0.18
Cardiac agents, n (%)	71 (1.6)	53 (1.7)	18 (1.5)	0.84
QT prolonging drugs, n (%)	4 (0.1)	2 (0.1)	2 (0.2)	0.64
*APOE* ε4 allele, n (%)	689 (15.9)	492 (15.6)	197 (16.8)	0.35
Resting heart rate, bpm	67.23 (10.9)	66.90 (10.7)	68.10 (11.5)	0.004
QT interval length, ms	396.91 (32.9)	396.59 (32.9)	397.75 (33.1)	0.347
QTc interval length, ms	417.06 (28.3)	415.80 (28.2)	420.44 (28.3)	< 0.001
JT interval length, ms	299.35 (33.7)	298.69 (33.8)	301.12 (33.5)	0.03
JTc interval length, ms	314.18 (28.3)	314.18 (28.3)	314.18 (28.3)	< 0.001
QRS interval length, ms	97.56 (12.8)	97.90 (12.5)	96.63 (13.3)	< 0.001
Total intracranial volume, ml ^†^	1361.46 (145.2)	1367.78 (144.5)	1341.32 (146.2)	0.007
Gray matter volume, ml ^†^	523.20 (50.6)	526.85 (50.6)	511.56 (48.8)	< 0.001
White matter volume, ml ^†^	446.74 (53.0)	450.30 (52.5)	435.40 (52.9)	< 0.001
Cerebrospinal fluid volume, ml ^†^	386.42 (103.9)	385.83 (103.0)	388.29 (106.8)	0.91
WMH volume, ml ^†^	8.95 (11.3)	8.39 (10.8)	10.71 (12.3)	0.007
Peripheral WMH volume, ml ^†^	7.60 (10.6)	7.07 (10.2)	9.30 (11.7)	0.004
Deep WMH volume, ml ^†^	1.35 (1.4)	1.32 (1.4)	1.41 (1.4)	0.27

Data are mean (standard deviation), unless otherwise specified. SBP systolic blood pressure; DBP, Diastolic blood pressure; TC, Total cholesterol; eGFR, Estimated glomerular filtration rate; BMI, Body mass index; TIA, Transient ischemic attack; *APOE*, Apolipoprotein E gene; WMH, White matter hyperintensities.

* The number of participants with missing values was 6 for SBP and 6 for DBP.

^†^ Data on brain MRI measures were available only in the brain MRI subsample (n = 989).

In the MRI subsample, 236 (23.9%) of the 989 participants were defined with MCI. Individuals with MCI (vs. no MCI) had lower volumes of ICV, GM, and WM and greater volumes of total WMH and PWMH (*p* < 0.01), while people without and with MCI did not differ significantly in CSF and DWMH volumes (Table 1).

### *Association of ventricular ECG parameters with MCI, aMCI, and naMCI (n* = *4328)*

Logistic regression analysis suggested that longer QT, QTc, JT, and JTc intervals were significantly associated with an increased likelihood of MCI after adjusting for a wide range of demographic, lifestyle, and clinical factors (all *p* < 0.05, Model 2, Table 2). Similar results were obtained for the associations of QT, QTc, JT, and JTc intervals with aMCI, and the multivariable-adjusted odds ratio of aMCI associated with per 1-SD increment in QT interval was 1.13 (95% confidence interval [CI]: 1.03, 1.25), while there was no significant association of ECG parameters with naMCI. In addition, QRS interval was not significantly associated with MCI, aMCI, or naMCI (Table 2). In the sensitivity analysis, we repeated the analysis among participants who were free from atrial fibrillation or stroke. The findings remained generally consistent (Supplementary Tables 1 and 2).

**Table 2:** Association of ventricular electrocardiogram parameters with mild cognitive impairment and its subtypes (n = 4328).

ECG parameters (per 1-SD increment)	Model 1 *		Model 2 *	
	Odds ratio (95% CI)	*P* value	Odds ratio (95% CI)	*P* value
MCI (n = 1,172)				
QT interval	**1.10 (1.00, 1.21)**	**0.05**	**1.10 (1.00, 1.21)**	**0.05**
QTc interval	**1.08 (1.01, 1.17)**	**0.03**	**1.09 (1.01, 1.17)**	**0.03**
JT interval	**1.11 (1.01, 1.22)**	**0.03**	**1.11 (1.01, 1.22)**	**0.03**
JTc interval	**1.09 (1.01, 1.17)**	**0.03**	**1.09 (1.01, 1.17)**	**0.03**
QRS interval	0.98 (0.91, 1.05)	0.53	0.98 (0.91, 1.06)	0.61
aMCI (n = 991)				
QT interval	**1.13 (1.02, 1.25)**	**0.02**	**1.13 (1.03, 1.25)**	**0.01**
QTc interval	**1.10 (1.01, 1.19)**	**0.02**	**1.10 (1.02, 1.19)**	**0.02**
JT interval	**1.14 (1.03, 1.26)**	**0.01**	**1.14 (1.03, 1.26)**	**0.01**
JTc interval	**1.10 (1.02, 1.20)**	**0.02**	**1.10 (1.02, 1.20)**	**0.02**
QRS interval	0.99 (0.91, 1.06)	0.71	0.99 (0.92, 1.07)	0.85
naMCI (n = 181)				
QT interval	0.94 (0.78, 1.15)	0.56	0.90 (0.74, 1.10)	0.27
QTc interval	1.02 (0.87, 1.19)	0.83	0.99 (0.84, 1.16)	0.87
JT interval	0.99 (0.81, 1.21)	0.90	0.96 (0.79, 1.18)	0.67
JTc interval	1.01 (0.86, 1.20)	0.87	0.99 (0.85, 1.18)	0.94
QRS interval	0.92 (0.77, 1.09)	0.34	0.88 (0.73, 1.05)	0.17

SD, Standard deviation; MCI, Mild cognitive impairment; aMCI, Amnestic mild cognitive impairment; naMCI, Non-amnestic mild cognitive impairment; ECG, Electrocardiogram; CI, Confidence interval.

* Model 1 was adjusted for age, sex, education, and heart rate (QT, JT, and QRS interval only); Model 2 was additionally adjusted for smoking, alcohol intake, body mass index, dyslipidemia, hypertension, diabetes, *APOE* genotype, coronary heart disease, arrhythmia, heart failure, stroke, transient ischemic attack, and use of anti-thrombotic agents, cardiac agents, and QT prolonging agents.

### *Association of ventricular ECG parameters with structural brain measures (n* = *989)*

Longer QT interval was significantly associated with lower GM volume, with the multivariable-adjusted β coefficient (per 1-SD increment in QT interval) being -2.77 (95% CI: -5.23, -0.30) (Table 3). Besides, prolonged JT and JTc interval were marginally associated with decreased WM volume. Furthermore, prolonged QT, QTc, JT, and JTc intervals were significantly associated with increased WMH volume, with the β coefficient of cubic-rooted WMH volume associated with per 1-SD increment in QT interval and JT interval being 0.08 (0.03, 0.13, *p* = 0.002) and 0.06 (0.01, 0.12, *p* = 0.02), respectively. Analogous patterns were obtained for the association of ECG parameters with PWMH volume, but there was no significant association with DWMH volume in the multivariable-adjusted model. In addition, QRS interval was not related to any of the examined structural brain MRI measures (Table 3). The sensitivity analysis among participants who were from atrial fibrillation or stroke yielded the results that were overall similar to those from the main analysis (Supplementary Tables 3 and 4).

**Table 3:** Association of ventricular electrocardiogram parameters with structural brain MRI measures (n = 989).

ECG parameters (per 1-SD increment)	Model 1 *		Model 2 *	
	β coefficient (95% CI), MRI measures	*P* value	β coefficient (95% CI), MRI measures	*P* value
GM volume, ml				
QT interval	**− 2.84 (− 5.29, − 0.39)**	**0.02**	**− 2.77 (− 5.23, − 0.30)**	**0.03**
QTc interval	**− 2.05 (− 4.06, − 0.04)**	**0.05**	**− **1.94 (**− **3.95, 0.08)	0.06
JT interval	**− **2.04 (**− **4.48, 0.41)	0.10	**− **1.97 (**− **4.41, 0.48)	0.11
JTc interval	**− **1.38 (**− **3.45, 0.68)	0.19	**− **1.32 (**− **3.39, 0.74)	0.21
QRS interval	**− **1.29 (**− **3.31, 0.72)	0.21	**− **1.27 (**− **3.32, 0.77)	0.22
WM volume, ml				
QT interval	**− **2.53 (**− **5.35, 0.28)	0.08	**− **2.13 (**− **4.94, 0.68)	0.14
QTc interval	**− **2.08 (**− **4.39, 0.22)	0.08	**− **1.73 (**− **4.03, 0.57)	0.14
JT interval	**− 3.21 (− 6.01, − 0.41)**	**0.02**	**− **2.75 (**− **5.53, 0.03)	0.05
JTc interval	**− 2.50 (− 4.87, − 0.14)**	**0.04**	**− **2.12 (**− **4.46, 0.23)	0.08
QRS interval	1.41 (**− **0.91, 3.72)	0.23	1.36 (**− **0.97, 3.68)	0.25
CSF volume, ml				
QT interval	4.44 (**− − **0.04, 8.93)	0.05	4.04 (**− **0.44, 8.53)	0.08
QTc interval	3.38 (**− **0.29, 7.05)	0.07	2.96 (**− **0.71, 6.63)	0.11
JT interval	4.46 (0.00, 8.93)	0.05	3.97 (**− **0.47, 8.42)	0.08
JTc interval	3.24 (**− **0.52, 7.01)	0.09	2.83 (**− **0.92, 6.57)	0.14
QRS interval	**− **0.32 (**− **4.01, 3.37)	0.86	**− **0.22 (**− **3.94, 3.50)	0.91
WMH volume, transformed^†^				
QT interval	**0.09 (0.04, 0.14)**	** < 0.001**	**0.08 (0.03, 0.13)**	**0.002**
QTc interval	**0.08 (0.04, 0.12)**	** < 0.001**	**0.07 (0.03, 0.11)**	**0.001**
JT interval	**0.07 (0.02, 0.12)**	**0.008**	**0.06 (0.01, 0.12)**	**0.02**
JTc interval	**0.06 (0.02, 0.10)**	**0.008**	**0.05 (0.01, 0.10)**	**0.01**
QRS interval	0.03 (**− **0.01, 0.08)	0.13	0.03 (**− **0.02, 0.07)	0.24
PWMH volume, transformed^†^				
QT interval	**0.09 (0.04, 0.14)**	** < 0.001**	**0.08 (0.03, 0.13)**	**0.003**
QTc interval	**0.08 (0.04, 0.12)**	** < 0.001**	**0.07 (0.03, 0.11)**	**0.001**
JT interval	**0.07 (0.02, 0.12)**	**0.008**	**0.06 (0.01, 0.12)**	**0.02**
JTc interval	**0.06 (0.02, 0.10)**	**0.008**	**0.05 (0.01, 0.10)**	**0.01**
QRS interval	0.03 (**− **0.01, 0.07)	0.17	0.02 (**− **0.02, 0.07)	0.27
DWMH volume, transformed^†^				
QT interval	**0.03 (0.00, 0.06)**	**0.04**	0.02 (**− **0.01, 0.05)	0.11
QTc interval	0.02 (0.00, 0.05)	0.05	0.02 (0.00, 0.04)	0.11
JT interval	0.01 (**− **0.01, 0.04)	0.31	0.01 (**− **0.02, 0.04)	0.42
JTc interval	0.01 (**− **0.01, 0.04)	0.27	0.01 (**− **0.01, 0.04)	0.33
QRS interval	**0.03 (0.00, 0.05)**	**0.03**	0.02 (0.00, 0.04)	0.11

ECG, electrocardiogram; CI, confidence interval; GM, grey matter; WM, white matter; CSF, cerebrospinal fluid; WMH, white matter hyperintensities; PWMH, periventricular white matter hyperintensities; DWMH, deep white matter hyperintensities.

* Model 1 was adjusted for age, sex, education, heart rate (QT, JT, and QRS interval only), MRI center, and ICV; Model 2 was additionally adjusted for smoking, alcohol intake, body mass index, dyslipidemia, hypertension, diabetes, *APOE* genotype, coronary heart disease, arrhythmia, heart failure, stroke, transient ischemic attack, and use of anti-thrombotic agents, cardiac agents, and QT prolonging agents.

^†^WMH, PWMH, and DWMH volume variables were cubic-root transformed to normalize the distributions.

**Figure 2:**
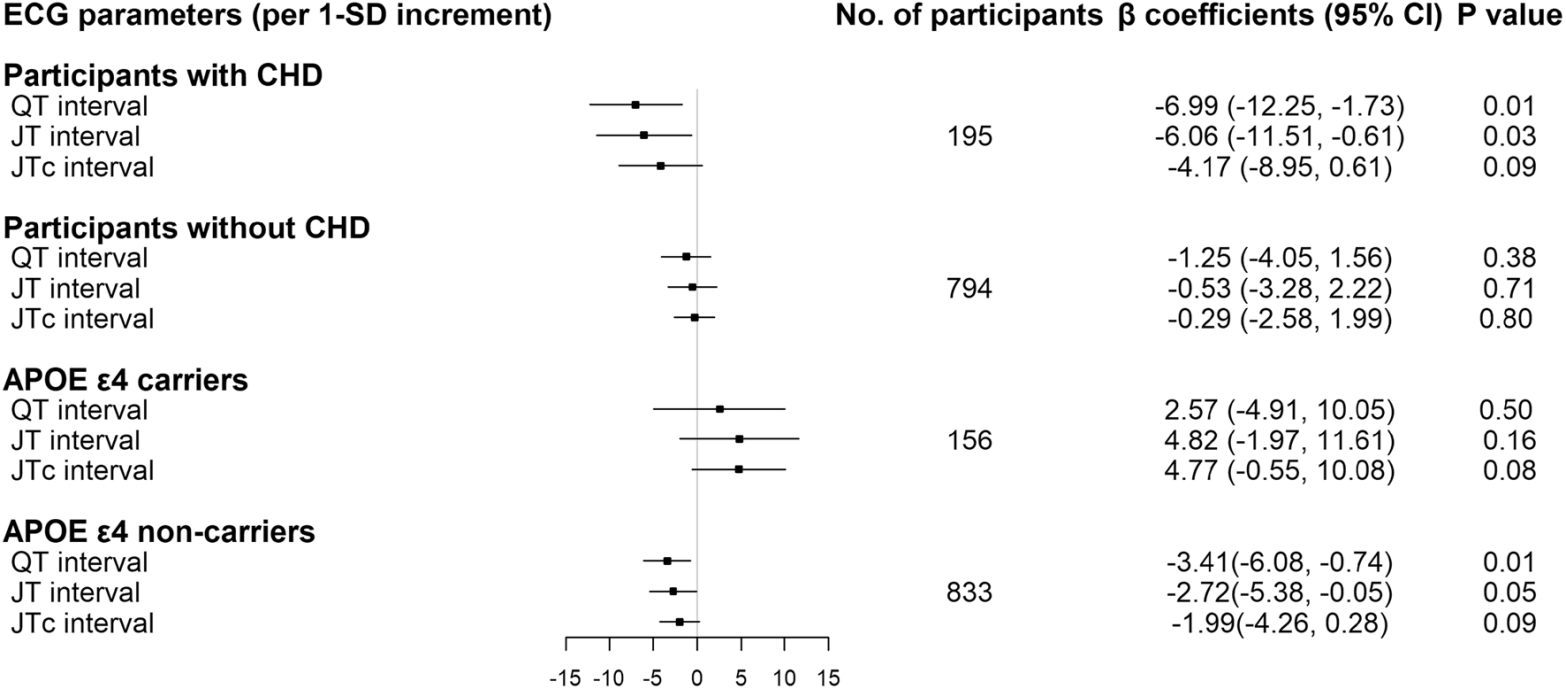
Association of QT, JT, and JTc intervals with grey matter volume stratified by history of CHD and *APOE* genotype (n = 989). CHD, Coronary heart diseases; CI, Confidence interval. Models were adjusted for age, sex, education, heart rate (QT and JT intervals only), MRI center, total intracranial volume, smoking, alcohol intake, body mass index, dyslipidemia, hypertension, diabetes, *APOE* genotype (if applicable), CHD (if applicable), arrhythmia, heart failure, stroke, transient ischemic attack, and use of anti-thrombotic agents, cardiac agents, and QT prolonging agents.

We detected statistical interactions of CHD and *APOE* ε4 allele with QT, JT, and JTc intervals on GM and CSF volumes (all *p* for interaction < 0.05). Stratified analysis showed prolonged QT interval was significantly associated with decreased GM volume and increased CSF volume among participants with CHD or those without *APOE* ε4 allele (*p* < 0.05), but not among those without the history of CHD or with *APOE* ε4 allele. The associations of GM volume with JT and JTc intervals stratified by history of CHD or *APOE* ε4 allele were overall comparable to those with QT interval (Fig. 2 and Supplementary Fig. 1).

## Discussion

In this cross-sectional study of rural-dwelling older adults in China, we investigated the association of ventricular ECG parameters with MCI, subtypes of MCI, and neuroimaging markers of brain lesions. Our main findings could be summarized as follows: (1) prolonged ventricular repolarization was independently associated with MCI, and aMCI in particular, but not with naMCI; (2) prolonged ventricular repolarization was independently associated with cortical atrophy and increased total WMH, and PWMH in particular, but not with DWMH; and (3) the associations of prolonged ventricular repolarization with atrophic brain measures varied by CHD and *APOE* gene, such that QT and JT intervals were associated with brain atrophy only among people with CHD or *APOE* ε4 non-carriers. Collectively, these results imply that prolonged ventricular repolarization is a valuable clinical marker for MCI, especially aMCI, and that altered ECG parameters and MCI may share common cerebral neuropathological lesions in a general population of rural older adults.

Previously, we reported that prolonged QT and JT intervals were associated with dementia and poor global cognitive function in older adults, even among those who were free of CVD^11,13^. Data from PROSPER study of older adults at high risk for cardiovascular events showed that prolonged ventricular repolarization was associated with poor function of several cognitive domains (e.g., attention and processing speed)^12^. Furthermore, other ventricular ECG parameters, such as the spatial QRS-T angle and left ventricular hypertrophy, have been associated with cognitive decline in older adults^14,15^. These studies support the view that abnormalities in ECG parameters is associated with cognitive phenotypes in older people. By contrast, participants in the Chicago Health and Aging Project (59% black) failed to show the association of QT prolongation with global cognitive performance, but other altered ventricular parameters (e.g., T wave nondipolar voltage) were associated with poor cognition^16^. Our current study revealed for the first time that prolonged ventricular repolarization was associated with MCI, and aMCI in particular, but not with naMCI, in a general population of rural older adults. Because individuals with aMCI were more likely to progress to dementia, especially AD, than those with naMCI^2,17^, these results imply that prolonged ventricular repolarization may be an early marker for prodromal dementia in older adults. Of note, the length of QRS interval was not associated with MCI and its subtypes in our study. Previous research has indicated that QRS prolongation, which is less common than QTc interval prolongation, was associated with increased long-term mortality^18^. Thus, the lack of cross-sectional association between QRS interval length and MCI in our study might be partially attributable to selective survival associated with QRS prolongation.

Evidence supporting the association of prolonged ventricular repolarization with brain MRI alterations is limited. The PROSPER study did not show any association of prolonged ventricular repolarization with structural brain MRI markers^12^. A possible explanation was that the PROSPER participants were relatively healthy in cognitive function (MMSE score ≥ 24), and thus, they might have fewer brain lesions. Indeed, the load of WMH in our study sample was about 3 times greater compared to that of the PROSPER participants. Our study further revealed the associations of prolonged ventricular repolarization with reduced GM volume and increased volumes of WMH, and PWMH in particular. Indeed, PWMH, but not DWMH, were associated with accelerated cognitive decline^19^, progression from aMCI to AD dementia^20^, and an increased risk of dementia^21^. In addition, we revealed the CHD- and *APOE* gene-varying associations between QT prolongation and brain atrophy, suggesting that CVD may be involved in the association of ECG markers with brain atrophic lesions, and that *APOE* ε4 allele may share a different pathophysiological process. This merits further investigation in future studies. Collectively, these results support the view that neuropathological alterations (e.g., cerebral microvascular and atrophic lesions) may be the common pathophysiological process linking prolonged ventricular repolarization with cognitive phenotypes.

Several possible mechanisms may underlie the observed associations. First, prolonged ventricular repolarization may be linked with cerebral hypoperfusion, and thus affecting cognitive function. Indeed, large-scale prospective studies showed that QT prolongation was a risk factor for incident stroke^7,22^. Second, prolonged ventricular repolarization is known to be associated with CVD (e.g., CHD and heart failure)^7,23^, which might further lead to cognitive impairment. Third, previous studies from the US and our own project have indicated that prolonged ventricular repolarization were associated with elevated serum inflammatory cytokines (e.g., IL-6) and adhesion molecules (e.g., ICAM-1 and VCAM-1)^11,24^. Inflammatory cytokines could lead to long-QT syndrome either directly through membrane ion channels modulation or indirectly through the reduction of testosterone levels or bioavailability of QT-prolonging drugs^25,26^. Thus, systemic inflammation may play an important role in linking prolonged ventricular repolarization with WMH and MCI. Fourth, altered ECG parameters may be related to autonomic dysfunction, which was common in patients with MCI^27^. Previous research reported a correlation between QT prolongation and dorsal vagal nucleus infarction^28^, which might affect the parasympathetic pathway and dysregulate the automatic neural control of cardiac function^29^. This indicates that prolonged ventricular repolarization may be cerebrogenic arrhythmia. Finally, given that WMH were associated with cerebral amyloid angiopathy and aMCI and AD^30,31^, prolonged ventricular repolarization could be linked with aMCI and AD partially via cerebral microvascular lesions. Indeed, previous research has linked prolonged QT, JT, and JTc intervals with plasma AD-related biomarkers (e.g., a decreased Aβ42/Aβ40 ratio and increased plasma neurofilament light chain protein)^13^.

Our study showed that prolonged ventricular repolarization was associated with MCI, subtypes of MCI, and MRI markers of brain microvascular and atrophic lesions in older adults. Many medications could affect QT intervals, such as Alzheimer’s drugs (e.g., galantamine and donepezil), cardiovascular agents (e.g., amiodarone and nicardipine), and antipsychotic agents (e.g., clozapine, escitalopram, and amantadine)^32,33^. Besides, previous studies showed that QT prolongation was associated with an increased risk of stroke and cardiac mortality^22,34^. Therefore, it is of clinical importance to recognize the prolonged QT intervals for older adults when taking these medications, and cognitive assessments and brain MRI examination may be considered for older adults with prolonged QT intervals.

This large-scale study engaged rural-dwelling Chinese older adults who had relatively low income and received no or very limited education (83.1% had no formal school education or only attended primary school). Given that most studies of this topic in the current literature have been carried out among urban populations in high-income countries, findings from our study could contribute to health equity across ethnically, socioeconomically, and demographically diverse populations. In addition, by integrating structural brain MRI data with extensive epidemiological, clinical, neuropsychological, and pharmacological profiles, we were able to explore the underlying neuropathologies linking prolonged ventricular repolarization with MCI and subtypes of MCI. However, our study also has limitations. First, we were not able to determine the causality due to the nature of cross-sectional study and the cross-sectional design also limited the investigation of the possible mechanisms underlying the observed associations and the interpretation of the research findings. Future prospective longitudinal studies are warranted to help clarify the potential causal relationship of prolonged ventricular repolarization with cognitive phenotypes and the underlying neuropathological mechanisms. Second, although we have controlled for extensive possible confounders, we cannot rule out the potential impact of residual confounding due to imperfect measurements of some confounders (e.g., self-reported lifestyle factors and health history). Finally, participants in the MRI subsample were younger, healthier, and more educated compared to those in the MIND-China total sample^35^. This should be kept in mind when generalizing these findings to other populations.

Our cross-sectional study revealed associations of prolonged ventricular repolarization with MCI, especially aMCI, but not with naMCI, in older adults. Furthermore, altered ECG parameters were associated with reduced GM volume and increased WMH volume, and increased PWMH volume in particular, but not with DWMH volume. The associations of ECG parameters with structural brain MRI markers varied by CHD and *APOE* gene, such that prolonged QT and JTs interval were associated with brain atrophy only among individuals who had CHD or did not carry the *APOE* ε4 allele. These findings suggest that prolonged ventricular repolarization is a valuable marker for prodromal dementia and vascular brain pathology in older adults, and that cognitive assessment and brain MRI examination may be important for older adults with QT prolongation.

## Methods

### Study design and participants

This is a cross-sectional study. We used data collected from the baseline assessments of the Multimodal INterventions to delay Dementia and disability in rural China (MIND-China) project, which is part of the World-Wide FINGERS Network^36,37^. MIND-China targeted at people who were aged ≥ 60 years by the end of 2017 and living in the 52 villages of Yanlou town, Yanggu County, western Shandong province. In March-September 2018, 5,765 participants (74.9% of all eligible persons) underwent the baseline assessments. Participants who were aged 60–64 years (n = 519) were excluded because a considerable proportion of people in this age group were working as rural migrant workers, and thus they could not attend the assessments. Of the 5,246 participants who were aged ≥ 65 years, we further excluded 918 persons due to prevalent dementia (n = 302), severe mental health problems (e.g., depressive symptoms and schizophrenia, n = 46), and missing information on MCI diagnosis (n = 329), ECG parameters (n = 12), or covariates (n = 229), leaving 4328 participants for the analysis of ECG parameters in relation to MCI (analytical sample 1). Using the cluster (village)-randomized sampling approach, a subsample of 1,034 participants were selected from 26 villages and invited for structural brain magnetic resonance imaging (MRI) scans, of these, 45 persons were excluded due to suboptimal MRI quality, leaving 989 individuals for the analysis involving MRI markers (analytical sample 2). Figure 1 shows the flowchart of the study participants.

The Ethics Committee at Shandong Provincial Hospital approved the protocol of MIND-China study. Prior to data collection, written informed consent was obtained from all participants or their guardians in the case of persons with severe cognitive impairment. The MIND-China study was registered in the Chinese Clinical Trial Registry (registration no.: ChiCTR1800017758). Research within MIND-China has been conducted in full compliance with the ethical principles expressed in the Declaration of Helsinki.

### Data collection and assessments

Data were collected following the structured questionnaire via face-to-face interviews, clinical and neurological examination, neuropsychological testing, and laboratory tests, as previously described^36^. In brief, we collected data on demographic features (age, sex, and education), lifestyle factors (e.g., smoking and alcohol consumption), genetic markers (e.g., *APOE* gene), health history (e.g., body mass index [BMI], dyslipidemia, hypertension, diabetes, CHD, arrhythmia, heart failure, stroke, and transient ischemic attacks [TIAs]), and administered drugs (anti-thrombotic agents, cardiac agents, and QT prolonging agents), which were considered potential confounders. Education was categorized into no formal education, primary school (1–5 years), and middle school or above (≥ 6 years). Smoking and alcohol intake were categorized into never, former, and current smoking or drinking alcohol. Apolipoprotein E (*APOE*) genotype was dichotomized into carriers vs. non-carriers of the ε4 allele. Information on the current use of medications was collected via in-person interviews, and whenever available, drug prescriptions and containers were checked to verify the information. All medications were classified and coded according to the Anatomical Therapeutic Chemical (ATC) classification system^38^. Diabetes mellitus, dyslipidemia, and hypertension were defined by integrating self-reported history of respective disorders, clinical examination, blood tests (i.e., fasting blood glucose and serum lipids), and current use of respective mediations (i.e., antihypertensive, blood glucose-lowering, and lipids-lowering drugs), as previously described^36^. BMI, CHD, heart failure, stroke, TIAs, and arrhythmia were ascertained following the approaches as previously reported^11,36^. Antithrombotic agents (e.g., acetylsalicylic acid, clopidogrel, and warfarin), cardiac agents (e.g., amiodarone, digoxin, propafenone, isosorbide mononitrate, glyceryl trinitrate, and trimetazidine), and QT prolonging drugs (e.g., alprazolam, olanzapine, perphenazine, sulpiride, and tamoxifen) were considered potential confounders because use of these drugs might affect ECG parameters and might be associated with cognitive impairment as well^4,32^.

Electrocardiogram (ECG) was recorded in a resting supine position using a 12-lead CM 300 electrocardiograph (Comen Corp., Shenzhen, Guangdong, China). Resting heart rate, QT interval, and QRS interval were obtained from an automated analysis program in the device. JT interval was defined as the length of QT interval minus QRS interval. QTc and JTc intervals were the heart rate-corrected QT and JT intervals, respectively, and were calculated using the Bazett’s formula:^39^ QTc = QT/(60/[heart rate])^1/2^; JTc = JT/(60/[heart rate])^1/2^.

### Diagnosis of MCI, aMCI, and naMCI

MCI was diagnosed following the Peterson’s criteria that were operationalized in the same way as used in the Mayo Clinic Study of Aging, as previously described in detail^37,40^. Briefly, the criteria for defining MCI included: (1) subjective cognitive concern by subjects or informants; (2) objective cognitive impairment evidenced in at least one of the four cognitive domains; (3) essentially preserved functional activities; and (4) absence of dementia diagnosed according to the DSM-IV criteria^41^. The final judgment was based on both neuropsychological test scores and a consensus agreement among neurologists specialized in cognitive disorders. MCI was further categorized into aMCI if the memory domain was impaired or naMCI if there was no impairment in memory function^40^.

### MRI data acquisition and processing

All eligible participants were scanned on either the Philips Ingenia 3.0 T MR scanner in Shandong Southwestern Lu Hospital (n = 896) or the Philips Archiva 3.0 T MR scanner in Liaocheng People's Hospital (n = 93). The detailed parameters and processing procedure of the core MRI sequences are described previously^36,42^. We spatially normalized and segmented T1-weighted images and then automatically estimated the total intracranial volume (ICV), volumes of grey matter (GM), white matter (WM), and cerebrospinal fluid (CSF) via the Computational Anatomy Toolbox running on MATLAB (http://dbm.neuro.uni-jena.de/cat12/). ICV and MRI center were considered as potential confounders in the analysis involving brain MRI metrics. T2-FLAIR images were processed in AccuBrain® (BrainNow Medical Technology Ltd., Shenzhen, Guangdong, China) to acquire the volume of WMH^42^. We further categorized WMH into periventricular WMH (PWMH) and deep WMH (DWMH) according to continuity to ventricles.

### Statistical analysis

We reported mean (standard deviation, SD) for continuous variables, and frequency (%) for categorical variables. We compared characteristics of the study participants by MCI status using chi-squared test for categorical variables and Mann–Whitney U test for continuous variables with skewed distribution. ECG parameters (i.e., QT, QTc, JT, JTc, and QRS intervals) were transformed into standardized z-score^12^. We used multinomial logistic regression models to estimate the associations of QT, QTc, JT, JTc, and QRS intervals with MCI, aMCI, and naMCI. In the MRI subsample, we investigated the associations of ventricular ECG parameters with structural brain MRI metrics using general linear regression models. Owing to right-skewed distribution, WMH, PWMH, and DWMH volume variables were cubic-root transformed^42^. Statistical interactions were assessed by simultaneously incorporating the independent variables and their cross-product term into the same model. When a statistical interaction was detected (*p* for interaction < 0.05), stratified analysis was further performed to assess the magnitude and direction of the interaction.

We reported the main results from two models: Model 1 was adjusted for age, sex, education, and resting heart rate (only for QT, JT, and QRS intervals); and Model 2 was additionally adjusted for smoking, alcohol intake, BMI, dyslipidemia, hypertension, diabetes, *APOE* genotype, CHD, arrhythmia, heart failure, stroke, TIA, anti-thrombotic agents, cardiac agents, and QT prolonging drugs.

R version 3.6.2 (R Core Team: R Project for Statistical Computing, Vienna, Austria. http://www.r-project.org) was used for all statistical analyses. A two-tailed *p* < 0.05 was considered statistically significant.

### Supplementary Information

Supplementary Information.

## Data Availability

The datasets used and/or analyzed during the current study are available from the corresponding author upon reasonable request.
