# Supplementary material for: Prolonged ventricular repolarization associated with mild cognitive impairment and white matter hyperintensities: a cross-sectional study
Source: Sci Rep. 2024 Jul 2;14:15162. doi: 10.1038/s41598-024-65364-y (PMC11219852; doi:10.1038/s41598-024-65364-y)
Supplement: Supplementary file 1 — Supplementary Information. [file 41598_2024_65364_MOESM1_ESM.docx]

**SUPPLEMENTARY MATERIAL**

**Prolonged ventricular repolarization associated with mild cognitive impairment and white matter hyperintensities: a cross-sectional study**

Ming Mao, MD,^1,2^ Yiran Wei, MD,^1^ Chaoqun Wang, MD,^3^ Xiaolei Han, MD, PhD,^1,2^ Rui Liu, MD, PhD,^1,2^ Yi Dong, MD, PhD,^1,2^ Lin Song, MD, PhD,^1,2^ Lin Cong, MD, PhD,^1,2^ Yongxiang Wang, MD, PhD,^1,2,4,5*^ Yifeng Du, MD, PhD,^1,2,3*^ Chengxuan Qiu, PhD^3,5^

^1^Department of Neurology, Shandong Provincial Hospital affiliated to Shandong First Medical University, Jinan, Shandong, 250021, P.R. China;

^2^Key Laboratory of Endocrine Glucose & Lipids Metabolism and Brain Aging in Shandong First Medical University, Ministry of Education of the People’s Republic of China, Jinan, Shandong, 250021, P.R. China;

^3^Department of Neurology, Shandong Provincial Hospital, Shandong University, Jinan, Shandong, 250021, P.R. China;

^4^Institute of Brain Science and Brain-inspired Research, Shandong First Medical University & Shandong Academy of Medical Sciences, Jinan, Shandong, 250021, P.R. China;

^5^Aging Research Center and Center for Alzheimer Research, Department of Neurobiology, Care Sciences and Society, Karolinska Institutet-Stockholm University, Stockholm, 17177, Sweden.

**CONTENTS**

**Supplementary files**

**Supplementary Figure 1.** Association of QT, JT, and JTc intervals with CSF volume by history of coronary heart disease and *APOE* genotype in the analytical sample 2 (n=989).

**Supplementary Table 1.** Association of ventricular electrocardiogram parameters with mild cognitive impairment and its subtypes in participants without atrial fibrillation (n=4261).

**Supplementary Table 2.** Association of ventricular electrocardiogram parameters with mild cognitive impairment and its subtypes in participants without stroke (n=3681).

**Supplementary Table 3.** Association of ventricular electrocardiogram parameters with structural brain MRI measures in participants without atrial fibrillation (n=982).

**Supplementary Table 4.** Association of ventricular electrocardiogram parameters with structural brain MRI measures in participants without stroke (n=860).

**
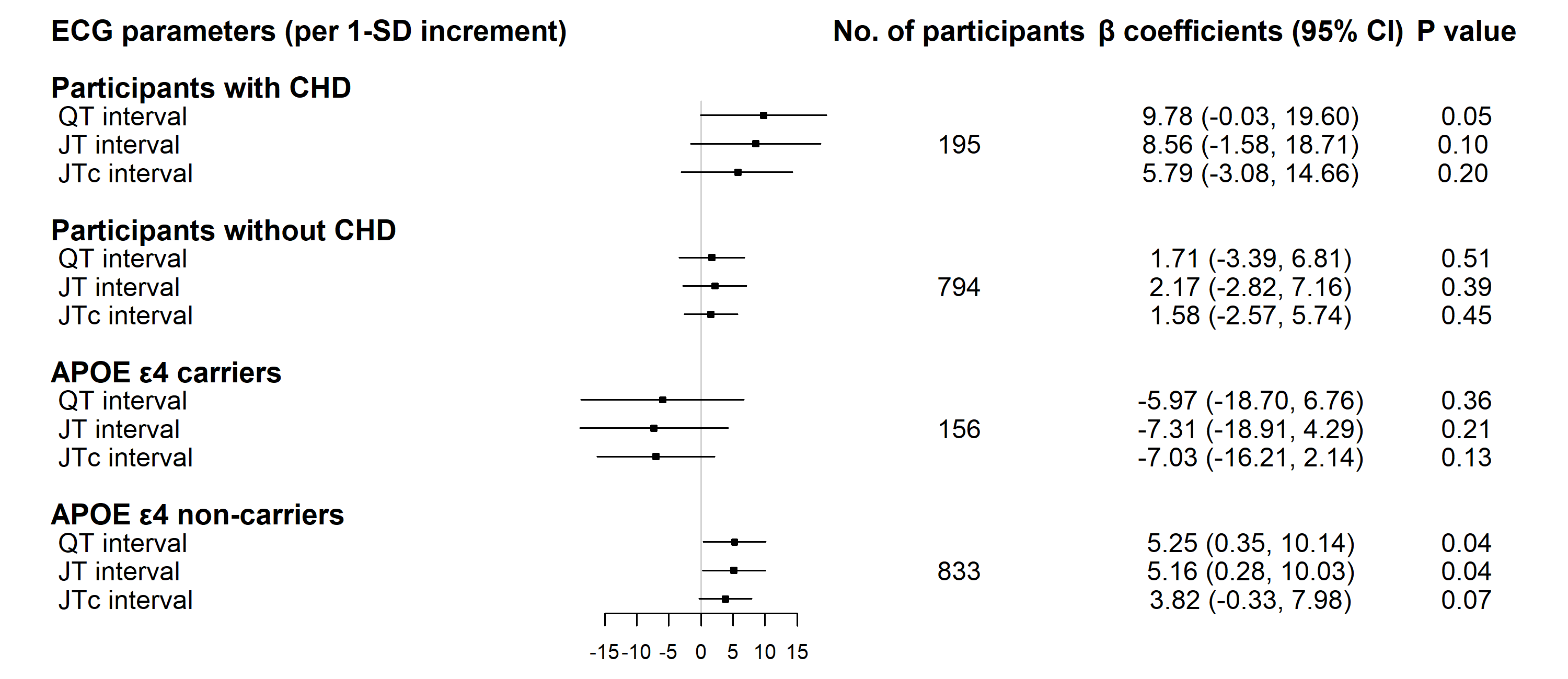
**

**Supplementary Figure 1.** **Association of QT, JT, and JTc intervals with CSF volume by history of coronary heart disease and *APOE* genotype in analytical sample 2 (n=989).**

Abbreviations: CSF, cerebrospinal fluid; CHD, coronary heart disease; *APOE*, apolipoprotein E gene; CI, confidence interval.

Models were adjusted for age, sex, education, heart rate (QT and JT intervals only), MRI center, total intracranial volume, smoking, alcohol intake, body mass index, dyslipidemia, hypertension, diabetes, *APOE* genotype (if applicable), CHD (if applicable), arrhythmia, heart failure, stroke, transient ischemic attack, and use of anti-thrombotic agents, cardiac agents, and QT prolonging agents.

**Supplementary Table 1. Association of ventricular electrocardiogram parameters with mild cognitive impairment and its subtypes in participants without atrial fibrillation (n=4261).**

| **ECG parameters (per 1-SD increment)** | **Model 1 ^*^** | |  | **Model 2 ^*^** | |
| --- | --- | --- | --- | --- | --- |
|  | **Odds ratio (95% CI)** | ***P* value** |  | **Odds ratio (95% CI)** | ***P* value** |
| **MCI (n=1,147)** | | | | | |
| QT interval | 1.09 (0.99, 1.20) | 0.07 |  | 1.09 (0.99, 1.20) | 0.08 |
| QTc interval | 1.07 (0.99, 1.15) | 0.07 |  | 1.07 (0.99, 1.15) | 0.08 |
| JT interval | **1.11 (1.01, 1.22)** | **0.03** |  | **1.10 (1.01, 1.22)** | **0.04** |
| JTc interval | **1.08 (1.00, 1.17)** | **0.05** |  | 1.08 (1.00, 1.17) | 0.05 |
| QRS interval | 0.97 (0.90, 1.04) | 0.43 |  | 0.97 (0.90, 1.05) | 0.50 |
| **aMCI (n=971)** | | | | | |
| QT interval | **1.12 (1.01, 1.24)** | **0.03** |  | **1.13 (1.02, 1.24)** | **0.02** |
| QTc interval | **1.08 (1.00, 1.17)** | **0.05** |  | **1.08 (1.00, 1.17)** | **0.04** |
| JT interval | **1.13 (1.02, 1.25)** | **0.02** |  | **1.13 (1.02, 1.25)** | **0.02** |
| JTc interval | **1.10 (1.01, 1.19)** | **0.03** |  | **1.10 (1.01, 1.19)** | **0.03** |
| QRS interval | 0.98 (0.91, 1.06) | 0.63 |  | 0.99 (0.91, 1.07) | 0.78 |
| **naMCI (n=176)** | | | | | |
| QT interval | 0.92 (0.76, 1.13) | 0.42 |  | 0.88 (0.73, 1.07) | 0.19 |
| QTc interval | 0.99 (0.84, 1.16) | 0.90 |  | 0.96 (0.82, 1.13) | 0.62 |
| JT interval | 0.98 (0.80, 1.20) | 0.83 |  | 0.95 (0.78, 1.17) | 0.62 |
| JTc interval | 0.99 (0.85, 1.18) | 0.95 |  | 0.98 (0.83, 1.16) | 0.78 |
| QRS interval | 0.90 (0.75, 1.06) | 0.23 |  | 0.86 (0.71, 1.02) | 0.10 |

Abbreviations: SD, standard deviation; MCI, mild cognitive impairment; aMCI, amnestic mild cognitive impairment; naMCI, non-amnestic mild cognitive impairment; ECG, electrocardiogram; CI, confidence interval.

^*^ Model 1 was adjusted for age, sex, education, and heart rate (QT, JT, and QRS interval only); Model 2 was additionally adjusted for smoking, alcohol intake, body mass index, dyslipidemia, hypertension, diabetes, *APOE* genotype, coronary heart disease, arrhythmia, heart failure, stroke, transient ischemic attack, and use of anti-thrombotic agents, cardiac agents, and QT prolonging agents.

**Supplementary Table 2. Association of ventricular electrocardiogram parameters with mild cognitive impairment and its subtypes in participants without stroke (n=3681).**

| **ECG parameters (per 1-SD increment)** | **Model 1 ^*^** | |  | **Model 2 ^*^** | |
| --- | --- | --- | --- | --- | --- |
|  | **Odds ratio (95% CI)** | ***P* value** |  | **Odds ratio (95% CI)** | ***P* value** |
| **MCI (n=956)** | | | | | |
| QT interval | **1.13 (1.02, 1.25)** | **0.02** |  | 1.13 (1.02, 1.26) | **0.02** |
| QTc interval | **1.10 (1.01, 1.19)** | **0.02** |  | 1.10 (1.01, 1.19) | **0.03** |
| JT interval | **1.12 (1.01, 1.24)** | **0.04** |  | 1.11 (1.00, 1.24) | **0.04** |
| JTc interval | 1.08 (1.00, 1.18) | 0.06 |  | 1.08 (0.99, 1.18) | 0.07 |
| QRS interval | 1.02 (0.94, 1.10) | 0.67 |  | 1.02 (0.95, 1.11) | 0.55 |
| **aMCI (n=850)** | | | | | |
| QT interval | 1.16 (1.04, 1.29) | 0.006 |  | 1.16 (1.04, 1.30) | 0.006 |
| QTc interval | 1.11 (1.02, 1.21) | 0.01 |  | 1.11 (1.02, 1.21) | 0.01 |
| JT interval | 1.14 (1.02, 1.27) | 0.02 |  | 1.14 (1.02, 1.27) | 0.02 |
| JTc interval | 1.10 (1.01, 1.20) | 0.03 |  | 1.10 (1.01, 1.20) | 0.03 |
| QRS interval | 1.02 (0.94, 1.11) | 0.59 |  | 1.03 (0.95, 1.12) | 0.47 |
| **naMCI (n=106)** | | | | | |
| QT interval | 0.90 (0.71, 1.17) | 0.42 |  | 0.91 (0.72, 1.18) | 0.46 |
| QTc interval | 0.96 (0.79, 1.18) | 0.68 |  | 0.97 (0.80, 1.19) | 0.74 |
| JT interval | 0.92 (0.73, 1.20) | 0.52 |  | 0.93 (0.74, 1.21) | 0.58 |
| JTc interval | 0.94 (0.77, 1.16) | 0.53 |  | 0.95 (0.78, 1.17) | 0.59 |
| QRS interval | 0.97 (0.77, 1.19) | 0.77 |  | 0.96 (0.76, 1.18) | 0.71 |

Abbreviations: SD, standard deviation; MCI, mild cognitive impairment; aMCI, amnestic mild cognitive impairment; naMCI, non-amnestic mild cognitive impairment; ECG, electrocardiogram; CI, confidence interval.

^*^ Model 1 was adjusted for age, sex, education, and heart rate (QT, JT, and QRS interval only); Model 2 was additionally adjusted for smoking, alcohol intake, body mass index, dyslipidemia, hypertension, diabetes, *APOE* genotype, coronary heart disease, arrhythmia, heart failure, transient ischemic attack, and use of anti-thrombotic agents, cardiac agents, and QT prolonging agents.

**Supplementary Table 3. Association of ventricular electrocardiogram parameters with structural brain MRI measures in participants without atrial fibrillation (n=982).**

| **ECG parameters (per 1-SD increment)** | **Model 1** ^*^ | |  | **Model 2** ^*^ | |
| --- | --- | --- | --- | --- | --- |
|  | **β coefficient (95% CI), MRI measures** | ***P* value** |  | **β coefficient (95% CI), MRI measures** | ***P* value** |
| **GM volume, ml** | | | | | |
| QT interval | **-2.97 (-5.44, -0.51)** | **0.02** |  | **-2.84 (-5.32, -0.36)** | **0.02** |
| QTc interval | **-2.17 (-4.19, -0.15)** | **0.04** |  | -1.99 (-4.02, 0.04) | 0.05 |
| JT interval | -2.03 (-4.48, 0.42) | 0.10 |  | -1.95 (-4.40, 0.50) | 0.12 |
| JTc interval | -1.34 (-3.41, 0.73) | 0.20 |  | -1.29 (-3.36, 0.78) | 0.22 |
| QRS interval | -1.51 (-3.54, 0.51) | 0.14 |  | -1.40 (-3.45, 0.65) | 0.18 |
| **WM volume, ml** | | | | | |
| QT interval | -2.72 (-5.55, 0.11) | 0.06 |  | -2.21 (-5.04, 0.63) | 0.13 |
| QTc interval | -2.24 (-4.56, 0.07) | 0.06 |  | -1.76 (-4.07, 0.56) | 0.14 |
| JT interval | **-3.30 (-6.11, -0.50)** | **0.02** |  | -2.86 (-5.65, -0.06) | **0.05** |
| JTc interval | **-2.56 (-4.93, -0.19)** | **0.03** |  | -2.21 (-4.57, 0.15) | 0.07 |
| QRS interval | 1.28 (-1.04, 3.60) | 0.28 |  | 1.44 (-0.90, 3.78) | 0.23 |
| **CSF volume, ml** | | | | | |
| QT interval | **4.74 (0.23, 9.25)** | **0.04** |  | 4.16 (-0.36, 8.69) | 0.07 |
| QTc interval | 3.63 (-0.06, 7.33) | 0.05 |  | 3.01 (-0.69, 6.71) | 0.11 |
| JT interval | **4.54 (0.06, 9.02)** | **0.05** |  | 4.06 (-0.40, 8.52) | 0.07 |
| JTc interval | 3.25 (-0.53, 7.04) | 0.09 |  | 2.88 (-0.88, 6.65) | 0.13 |
| QRS interval | 0.02 (-3.69, 3.72) | 0.99 |  | -0.20 (-3.94, 3.53) | 0.91 |
| **WMH volume, transformed**^†^ | | | | | |
| QT interval | **0.09 (0.04, 0.15)** | **<0.001** |  | **0.08 (0.03, 0.14)** | **0.002** |
| QTc interval | **0.08 (0.04, 0.12)** | **<0.001** |  | **0.07 (0.03, 0.12)** | **<0.001** |
| JT interval | **0.07 (0.02, 0.12)** | **0.007** |  | **0.06 (0.01, 0.12)** | **0.01** |
| JTc interval | **0.06 (0.02, 0.10)** | **0.008** |  | **0.05 (0.01, 0.10)** | **0.01** |
| QRS interval | 0.03 (-0.01, 0.08) | 0.13 |  | 0.03 (-0.02, 0.07) | 0.21 |
| **PWMH volume, transformed**^†^ | | | | | |
| QT interval | **0.09 (0.04, 0.15)** | **<0.001** |  | **0.08 (0.03, 0.14)** | **0.001** |
| QTc interval | **0.08 (0.04, 0.12)** | **<0.001** |  | **0.07 (0.03, 0.12)** | **<0.001** |
| JT interval | **0.07 (0.02, 0.13)** | **0.007** |  | **0.07 (0.01, 0.12)** | **0.01** |
| JTc interval | **0.06 (0.02, 0.10)** | **0.008** |  | **0.05 (0.01, 0.10)** | **0.01** |
| QRS interval | 0.03 (-0.01, 0.07) | 0.16 |  | 0.03 (-0.02, 0.07) | 0.23 |
| **DWMH volume, transformed**^†^ | | | | | |
| QT interval | **0.03 (0.00, 0.06)** | **0.05** |  | 0.02 (-0.01, 0.05) | 0.13 |
| QTc interval | 0.02 (0.00, 0.05) | 0.06 |  | 0.02 (0.00, 0.04) | 0.12 |
| JT interval | 0.01 (-0.01, 0.04) | 0.33 |  | 0.01 (-0.02, 0.04) | 0.45 |
| JTc interval | 0.01 (-0.01, 0.04) | 0.28 |  | 0.01 (-0.01, 0.04) | 0.35 |
| QRS interval | **0.03 (0.00, 0.05)** | **0.03** |  | 0.02 (0.00, 0.04) | 0.10 |

Abbreviations: ECG, electrocardiogram; CI, confidence interval; GM, grey matter; WM, white matter; CSF, cerebrospinal fluid; WMH, white matter hyperintensities; PWMH, periventricular white matter hyperintensities; DWMH, deep white matter hyperintensities.

^*^ Model 1 was adjusted for age, sex, education, heart rate (QT, JT, and QRS interval only), MRI center, and ICV; Model 2 was additionally adjusted for smoking, alcohol intake, body mass index, dyslipidemia, hypertension, diabetes, *APOE* genotype, coronary heart disease, arrhythmia, heart failure, stroke, transient ischemic attack, and use of anti-thrombotic agents, cardiac agents, and QT prolonging agents.

^†^WMH, PWMH, and DWMH volume variables were cubic-root transformed to normalize the distributions.

**Supplementary Table 4. Association of ventricular electrocardiogram parameters with structural brain MRI measures in participants without stroke (n=860).**

| **ECG parameters (per 1-SD increment)** | **Model 1** ^*^ | |  | **Model 2** ^*^ | |
| --- | --- | --- | --- | --- | --- |
|  | **β coefficient (95% CI), MRI measures** | ***P* value** |  | **β coefficient (95% CI), MRI measures** | ***P* value** |
| **GM volume, ml** | | | | | |
| QT interval | **-2.65 (-5.27, -0.03)** | **0.05** |  | **-2.66 (-5.29, -0.02)** | **0.05** |
| QTc interval | **-1.85 (-4.00, 0.29)** | 0.09 |  | -1.78 (-3.93, 0.38) | 0.11 |
| JT interval | -1.90 (-4.51, 0.72) | 0.15 |  | -1.82 (-4.43, 0.80) | 0.17 |
| JTc interval | -1.30 (-3.50, 0.90) | 0.25 |  | -1.25 (-3.45, 0.95) | 0.26 |
| QRS interval | -1.23 (-3.38, 0.92) | 0.26 |  | -1.36 (-3.53, 0.81) | 0.22 |
| **WM volume, ml** | | | | | |
| QT interval | -1.72 (-4.71, 1.26) | 0.26 |  | -1.55 (-4.54, 1.44) | 0.31 |
| QTc interval | -1.41 (-3.85, 1.04) | 0.26 |  | -1.17 (-3.62, 1.29) | 0.35 |
| JT interval | -2.29 (-5.26, 0.69) | 0.13 |  | -2.08 (-5.05, 0.89) | 0.17 |
| JTc interval | -1.74 (-4.25, 0.76) | 0.17 |  | -1.60 (-4.09, 0.90) | 0.21 |
| QRS interval | 1.12 (-1.33, 3.56) | 0.37 |  | 1.10 (-1.36, 3.56) | 0.38 |
| **CSF volume, ml** | | | | | |
| QT interval | 3.62 (-1.15, 8.39) | 0.14 |  | 3.48 (-1.31, 8.26) | 0.15 |
| QTc interval | 2.58 (-1.33, 6.50) | 0.20 |  | 2.29 (-1.63, 6.21) | 0.25 |
| JT interval | 3.59 (-1.17, 8.35) | 0.14 |  | 3.31 (-1.44, 8.05) | 0.17 |
| JTc interval | 2.54 (-1.47, 6.55) | 0.21 |  | 2.34 (-1.66, 6.33) | 0.25 |
| QRS interval | -0.14 (-4.06, 3.77) | 0.94 |  | 0.06 (-3.89, 4.00) | 0.98 |
| **WMH volume, transformed**^†^ | | | | | |
| QT interval | **0.08 (0.02, 0.13)** | **0.005** |  | **0.07 (0.02, 0.13)** | **0.01** |
| QTc interval | **0.07 (0.02, 0.11)** | **0.003** |  | **0.06 (0.02, 0.11)** | **0.007** |
| JT interval | **0.06 (0.00, 0.11)** | **0.05** |  | 0.05 (0.00, 0.11) | 0.06 |
| JTc interval | 0.05 (0.00, 0.09) | 0.05 |  | 0.04 (0.00, 0.09) | 0.06 |
| QRS interval | 0.04 (-0.01, 0.08) | 0.09 |  | 0.03 (-0.01, 0.08) | 0.19 |
| **PWMH volume, transformed**^†^ | | | | | |
| QT interval | **0.08 (0.02, 0.13)** | **0.007** |  | **0.07 (0.01, 0.12)** | **0.01** |
| QTc interval | **0.07 (0.02, 0.11)** | **0.004** |  | **0.06 (0.02, 0.11)** | **0.007** |
| JT interval | 0.05 (0.00, 0.11) | 0.05 |  | 0.05 (0.00, 0.10) | 0.07 |
| JTc interval | 0.04 (0.00, 0.09) | 0.06 |  | 0.04 (0.00, 0.09) | 0.07 |
| QRS interval | 0.04 (-0.01, 0.08) | 0.12 |  | 0.03 (-0.02, 0.07) | 0.20 |
| **DWMH volume, transformed**^†^ | | | | | |
| QT interval | 0.03 (0.00, 0.06) | **0.04** |  | 0.03 (-0.01, 0.06) | 0.11 |
| QTc interval | 0.02 (0.00, 0.05) | 0.07 |  | 0.02 (-0.01, 0.04) | 0.15 |
| JT interval | 0.02 (-0.02, 0.05) | 0.34 |  | 0.01 (-0.02, 0.04) | 0.42 |
| JTc interval | 0.01 (-0.01, 0.04) | 0.33 |  | 0.01 (-0.01, 0.04) | 0.37 |
| QRS interval | 0.03 (0.00, 0.05) | **0.03** |  | 0.02 (0.00, 0.05) | 0.09 |

Abbreviations: ECG, electrocardiogram; CI, confidence interval; GM, grey matter; WM, white matter; CSF, cerebrospinal fluid; WMH, white matter hyperintensities; PWMH, periventricular white matter hyperintensities; DWMH, deep white matter hyperintensities.

^*^ Model 1 was adjusted for age, sex, education, heart rate (QT, JT, and QRS interval only), MRI center, and ICV; Model 2 was additionally adjusted for smoking, alcohol intake, body mass index, dyslipidemia, hypertension, diabetes, *APOE* genotype, coronary heart disease, arrhythmia, heart failure, transient ischemic attack, and use of anti-thrombotic agents, cardiac agents, and QT prolonging agents.

^†^WMH, PWMH, and DWMH volume variables were cubic-root transformed to normalize the distributions.
